# Pancreatic schwannoma mimicking pancreatic cystadenoma: A case report and literature review of the imaging features

**DOI:** 10.1097/MD.0000000000016095

**Published:** 2019-06-14

**Authors:** Shunda Wang, Cheng Xing, Huanwen Wu, Menghua Dai, Yupei Zhao

**Affiliations:** a Department of General Surgery; b Department of Pathology, Peking Union Medical College Hospital, Beijing, China.

**Keywords:** diagnosis, imaging, pancreatic schwannoma, resection, schwannoma

## Abstract

**Introduction:**

Schwannomas, also known as neurilemmoma, are benign neoplasms that originating from Schwann cells in peripheral nerve sheaths. The head, neck, and extremities are the most common sites; however, pancreatic schwannomas are rare neoplasms. Accurate preoperative diagnosis of these tumors is very tough because of pancreatic schwannomas usually mimicking other cystic tumors. Here we present a case of pancreatic schwannoma misdiagnosed as pancreatic cystadenoma.

**Patient concerns:**

We presented a rare case of a 55-year-old female admitted to our hospital for abdominal distension. The physical examination and results of laboratory testing reveal no abnormalities.

**Diagnosis:**

A computed tomography (CT) scan detected a hypodense 2.4 cm × 2.6 cm mass with a clear margin at the neck of the pancreas. Pancreatic cystadenoma was strongly suspected.

**Interventions:**

The patient underwent robotic distal pancreatectomy with splenectomy. The gross specimen showed a pale and solid mass with a capsule.

**Outcomes:**

Histological examination of the surgical specimen demonstrated a pancreatic schwannoma. Immunohistochemistry results were as follows: S-100 (+), CD117 (−), SMA (−), and Desmin (−). She was discharged on postoperative day 6 and no recurrence of the tumor happened during the 12-month follow-up.

**Conclusion:**

Precise preoperative diagnosis of pancreatic schwannomas is very difficult despite the application of multiple imaging modalities. Surgery is the most effective treatment for this rare disease and the final diagnosis usually relies on pathology. Following complete tumor removal, patients with pancreatic schwannomas generally have a good prognosis.

## 1 Introduction

Schwannomas are mesenchymal tumors originating from Schwann cells in peripheral nerve sheaths. Although almost every part of the human body can be involved, the most common locations are the head, neck, trunk, and extremities.^[1]^ However, pancreatic schwannomas are rare. To our knowledge, only 65 cases of pancreatic schwannoma have been reported in the English literature during the last 40 years.^[2]^

Pancreatic schwannomas usually affect adults with an equal sex distribution (range: 20–87 years) or race preference.^[3,4]^ Pancreatic schwannomas vary in size (1–20 cm) and location (45% head, 23% body, 22% tail, 10% uncinate).^[5]^ Schwannomas are generally encapsulated tumors, and most of them are benign, whereas the pathogenesis of the tumor remains unclear.^[2]^ Accurate preoperative diagnosis of these tumors is very difficult if patients have no history of von Recklinghausen disease. Pancreatic schwannomas can mimic the whole spectrum of cystic pancreatic lesions including: intraductal mucinous-papillary neoplasms, mucinous or serous cystic neoplasms and pancreatic pseudocysts.^[6]^ Cystic pancreatic lesions account for 10% to 15% of pancreatic cysts and 1% of all pancreatic neoplasms. Cystic tumors of the pancreas are increasingly discovered on cross-sectional imaging. All these tumors may be benign, of borderline malignancy, or malignant. Accurate diagnosis is very tough because of pancreatic schwannomas usually mimicking other cystic tumors. Surgery is the most common treatment for schwannomas, and patients usually have a good prognosis.^[1]^

## 2 Case report

Fifty-five female patients were admitted to hospital because of abdominal distension for nearly 6 months, denying abdominal pain, diarrhea, vomiting, anorexia, hematochezia, and other discomfort. She had no history of clinical pancreatitis and there was no sign of von Recklinghausen disease. The physical examination was unremarkable. Results of laboratory testing revealed normal tumor biomarker, amylase, and liver enzymes.

**Figure 1:**
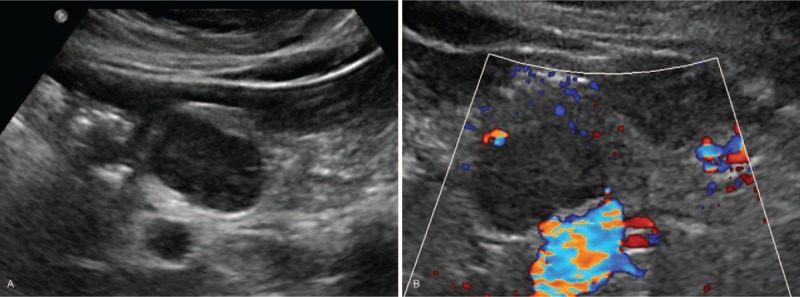
The 3.1 × 2.0 cm hypoechoic lesion with morphological rules and border clear at pancreatic body (A) without clear blood flow inside the signal (B).

She firstly received B ultrasound which revealed a 3.1 × 2.0 cm hypoechoic lesion with morphological rules and border clear at pancreatic body. There was no clear blood flow inside the signal. (Fig. 1) The echogenicity of the rest of the pancreas was homogenous, and pancreatic duct showed no obvious expansion. The result indicated the solid occupying at the body of the pancreas might be benign lesion, with high possibility of cystadenoma.

**Figure 2:**
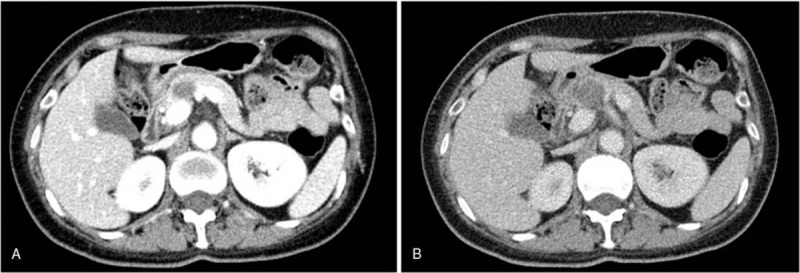
There was a 2.4 cm × 2.6 cm lesion with low density located at the neck of the pancreas on enhanced scan (A). Pancreatic duct showed no obvious expansion. Venous phase also manifested no obvious abnormalities (B).

To further define the character of the lesion, the patient underwent abdomen enhanced computer tomography (CT). The result indicated the round-like, clear edge lesion with low density located at the neck of the pancreas. The size of lesion was 2.4 cm × 2.6 cm, whereas it had the visible separation on enhanced scan. Pancreatic duct showed no obvious expansion. Abdominal artery and portal vein also manifested no obvious abnormalities. (Fig. 2) The first diagnosis from CT scan suggested the pancreatic neck lesion might be cystadenoma.

She underwent the laparoscopic exploration of the pancreas. The hard mass about 3 cm in diameter was seen at the upper part of the pancreatic body, part of it protruding the pancreas surface. We deduced the mass was in accordance with the performance of cystadenoma and decided to perform robotic distal pancreatectomy with splenectomy.

**Figure 3:**
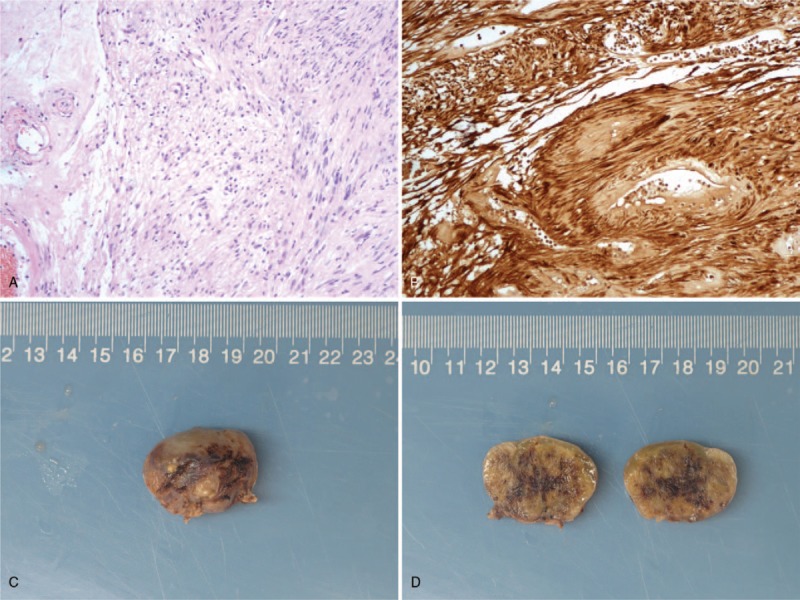
Photomicrograph showed spindle cells arranged in fascicular or whorled patterns. There was hypercellular and characterized by closely packed spindle cells with occasional nuclear palisading (A 100×). S-100 stain was positive (B 100×). The section of the tumor was pale and solid, and the tissue had clear boundary with capsule (C, D).

The approximately 2.0 × 2.0 × 1.8 cm mass with a capsule was resected. The section of the tumor was pale and solid, and the tissue had clear boundary with the surrounding tissue (Fig. 3). Microscopic examination showed that the tumor was mainly composed of spindle-shaped cells with palisading arrangement and no atypia, which is consistent with a benign tumor (Fig. 3). S-100 was commonly positive in schwannoma which was used as specific marker (Fig. 3). Pathological findings indicated the lesion was pancreatic schwannoma. Immunohistochemistry results were as follows: S-100 (+), CD117 (−), SMA (−), Desmin (−), DOG-1 (−), CD34 (+), SDHB (+) and Ki67 3%.

Postoperatively, the patient recovered well and left the hospital 6 days later with no evidence of postoperative pancreatic fistula. At a 12-month follow-up after resection, the patient is doing well without any recurrent.

## 3 Discussion

Schwannomas are benign neoplasms that originate in any nerve that has a Schwann cell sheath. The Schwann cell is the supporting element of the peripheral nerve and guides the regeneration of nerve fibers.^[7]^ More than 90% of schwannomas are benign but most with von Recklinghausen disease may be malignant schwannomas.^[8]^ Malignant schwannomas are uncommon and malignant transformation of a benign schwannoma is very rare.^[2]^ Malignant schwannomas were more likely to be larger-sized and the larger tumor size was related to cystic degeneration.^[6]^ Even though any part of the human body can be involved, the head, neck, and extremities are the most common sites.^[9]^ In the abdominal cavity, the retroperitoneum^[10]^ is the most common sites involved.

The main signs may be the tenderness of the upper abdomen and the laboratory data generally cannot help to indicate the specific diagnosis,^[11]^ such as the case in our report. In recent studies, approximately 70% of the patients are symptomatic at the time of diagnosis, and about 30% of the patients are asymptomatic, whereas the lesions are found incidentally on abdominal imaging.^[12]^ Even the patients with pancreatic cystadenoma, patients may not present with specific symptoms. It is a challenge to obtain a precise preoperative diagnosis, despite the application of multiple imaging modalities. The lack of radiological characteristics from other pancreatic lesions makes the preoperative diagnosis challenging. We summarize the different image features of each case in published literature,^[3,4,6–9,11–65]^ aiming to find the common manifestation which will be beneficial to diagnosis (Table 1).

**Table 1:**
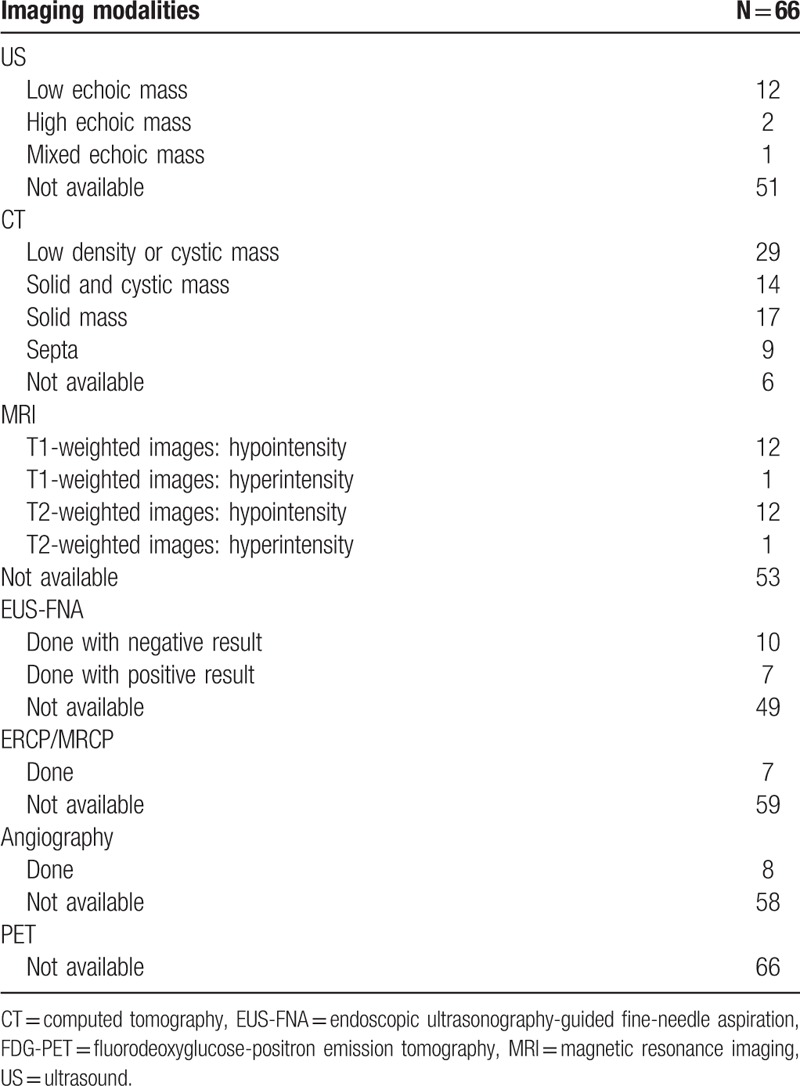
This table summarizes the imaging features among 66 cases of the pancreatic schwannoma including 65 cases of the literature review and the present case.

B-mode ultrasonography is not routinely used in screening pancreatic tumors. Pancreatic schwannoma is mostly hypoechoic in cases reported in the literature. Almost all benign pancreatic schwannomas are low density or cystic masses on normal CT scans. Sometimes pancreatic cystadenoma also manifests low density on CT scans. Contrast-enhanced CT findings of these tumors correlate quite well with pathological features and lesion size.^[19,29,64]^ Pancreatic schwannomas of Antoni type A show hypodense, solid masses with inhomogeneous or occasionally multiseptated enhancement. Those of Antoni type B show homogeneous cystic or multiseptated mass.^[11]^ The more vascular Antoni A areas are commonly enhanced, whereas Antoni B are nonenhanced.^[12]^ However, pancreatic cystadenoma usually appears as a lobulated hypodense mass, often with central coarse calcifications and fibrosis. After contrast injection, the central fibrous portions of the lesion enhance. The magnetic resonance imaging (MRI) findings of schwannomas in the pancreas have only been reported in 13 cases. A typical schwannoma appears hypointense in T1-weighted images and appears inhomogeneously hyperintense in T2-weighted images.^[6]^ However, pancreatic cystadenoma often shares those imaging features, and differential diagnoses should be considered.

Besides, the endoscopic ultrasonography-guided fine needle aspiration (EUS-FNA) may greatly contribute to precise preoperative diagnosis.^[18]^ There was a case reported that the pancreatic schwannoma was correctly diagnosed preoperatively by EUS-FNA.^[38]^ Sometimes it remains controversial because of its high false-negative rate. Although increased uptake of fluorodeoxyglucose in schwannomas of various sites has been reported,^[66,67]^ the usage for pancreatic schwannoma has never been reported. The usefulness of Fluorodeoxyglucose positron emission tomography (FDG-PET) for diagnosing pancreatic schwannoma would be investigated further. But for pancreatic cystadenoma, FDG-PET sometimes could be taken to discriminate the benign or low-grade malignant lesions. As for angiography, pancreatic schwannomas were reported to be either hypervascular or hypovascular. There were only 8 cases about angiography in imaging diagnosis of schwannomas because it was lacked of enhancement and unable to recognize minute vascularity.

The real challenge in the diagnosis of pancreatic schwannomas is the presence of cystic formation within the tumor.^[12]^ Lesions can often be misdiagnosed as benign pancreatic cysts. Therefore, definitive diagnosis can be achieved only based on the immunohistochemical examinations. Microscopically, a typical schwannoma is composed of 2 areas, namely Antoni A and Antoni B areas. The Antoni A area is hypercellular and characterized by closely packed spindle cells with occasional nuclear palisading. On the contrary, the Antoni B area is hypocellular and is occupied by loosely arranged tumor cells.^[60]^ Grossly, majority of pancreatic cystadenomas are well circumscribed, and the cut surface exhibits numerous cystic spaces filled with clear serous fluid. The solid variant appears as a well-circumscribed solid nodule lacking any grossly visible cystic spaces.

Surgery is the most common treatment for schwannomas. In this case, patient underwent distal pancreatectomy with splenectomy. Complete local excision is thought to be adequate surgical treatment for schwannomas. However, radical operations such as pancreatoduodenectomy were performed in some patients because a definite preoperative diagnosis could not be established. Intraoperative consultation with a pathologist may help surgeon to select the appropriate operative method, limiting it to enucleation, as opposed to radical resection.^[7]^

Following complete tumor excision, patients with pancreatic schwannomas generally have a good prognosis. Radiotherapy could be used in the future for the treatment of pancreatic schwannoma in patients that are not suitable for surgical resection.^[42]^ Because there is not clear evidence of the behavior of pancreatic schwannomas after resection, close radiological follow-up is recommended after surgical excision.^[12]^

## 4 Conclusion

In conclusion, the pancreatic schwannoma is rare. Precise preoperative diagnosis is very difficult despite the application of multiple imaging modalities. Pancreatic schwannomas deserve attention regarding the differential diagnosis of pancreatic cystic lesions. Surgery is the most effective treatment for pancreatic schwannoma. Simple enucleation is adequate if this is possible to achieve. Intraoperative frozen section is useful to diagnose schwannoma. Following complete tumor removal, patients with pancreatic schwannomas generally have a good prognosis.

## Author contributions

Wang SD designed the report; Xing Cheng contributed to literature search, review of the studies and data extractions; Wang SD contributed to verify the data analysis and scrutinized data; Wu HW prepared the photo and proofread the pathologic materials. Dai MH and Zhao YP contributed to supervision throughout the study. All the authors contributed to writing the manuscript. All authors approved the final version of the manuscript.

**Conceptualization:** Shunda Wang.

**Data curation:** Cheng Xing.

**Formal analysis:** Shunda Wang.

**Investigation:** Huanwen Wu.

**Software:** Cheng Xing.

**Supervision:** Cheng Xing, Menghua Dai.

**Visualization:** Huanwen Wu, Yupei Zhao.

**Writing – original draft:** Shunda Wang.

**Writing – review & editing:** Menghua Dai.

Menghua Dai orcid: 0000-0003-0640-1238.
